# On Antimoniated Ointment

**Published:** 1826-07

**Authors:** 


19. *Antimoniated Ointment.* This application is now becoming very general, as a counter-irritant, and we believe (with Dr. Fulton, of Leamington, who writes to us on the subject,) that the formula first given by Dr. Jenner, is the best. It is as follows: ung. catacei ʒix. ant. tart. ʒij. sacchari albi ʒj. hyd. sulph. rub. gr. v. ♏ ft. unguentum. Of late we have found a plaister much more convenient and speedy in its operation. Thus, a drachm of tartarized antimony incorporated with two drachms of common cerate, and spread on lint, will produce pimples in 24 or 36 hours. Or the irritant may be incorporated with Burgundy pitch or other plaister, in any proportion the practitioner pleases, adapted to the urgency of the case. There is no trouble to the patient, and he knows nothing of what is going on till the eruption begins to come out. He then complains lustily; but generally acknowledges that the plaister has relieved the internal pain or other affection.

